# Prognostic Significance of Preoperative NLR, MLR, and PLR Values in Predicting the Outcome of Primary Cytoreductive Surgery in Serous Epithelial Ovarian Cancer

**DOI:** 10.3390/diagnostics13132268

**Published:** 2023-07-04

**Authors:** Anna Rebeka Kovács, Anita Sulina, Kincső Sára Kovács, Luca Lukács, Péter Török, Rudolf Lampé

**Affiliations:** Department of Obstetrics and Gynecology, Faculty of Medicine, University of Debrecen, 98. Nagyerdei krt., 4032 Debrecen, Hungary

**Keywords:** epithelial ovarian cancer, cytoreductive surgery, prognostic factors

## Abstract

(1) The degree of cytoreduction achieved during primary debulking surgery (PDS) is an important prognostic factor for the survival of patients with epithelial ovarian cancer (EOC). Our aim was to investigate the prognostic value of preoperative laboratory parameters for the outcome of PDS. (2) We analyzed the preoperative laboratory parameters of 150 serous EOC patients who underwent PDS between 2006 and 2013. Receiver operating characteristic (ROC) curve analysis was used to determine the optimal cut-off values of the variables for predicting the PDS outcome. We used binary logistic regression to examine the independent predictive value of the factors for incomplete cytoreduction. (3) Among the parameters, we established optimal cut-off values for cancer antigen (Ca)-125, neutrophil-to-lymphocyte ratio (NLR), monocyte-to-lymphocyte ratio (MLR), and platelet-to-lymphocyte ratio (PLR) to predict the outcome of PDS. The results of binary logistic regression showed that stage (FIGO III-IV), MLR (>0.305), and Ca-125 (>169.15 kU/L) were independent significant predictors of the degree of tumor reduction achieved during PDS. (4) In the future, MLR, especially in combination with other parameters, may be useful in determining prognosis and selecting the best treatment option (PDS or neoadjuvant chemotherapy + interval debulking surgery) for ovarian cancer patients.

## 1. Introduction

Ovarian cancer is the second most common type of gynecologic malignancy, and still the fifth leading cause of cancer mortality among women [1]. With around 314,000 newly diagnosed cases, ovarian cancer was responsible for the death of around 207,000 patients worldwide in 2020 [2].

The histological presentation of ovarian malignancies varies from epithelial tumors of different histological types (serous, mucinous, endometrioid, clear cell, transitional cell, and mixed epithelial-stromal) to sex cord-stromal tumors, germ cell tumors, soft tissue tumors, unclassified type, and metastatic secondary tumors from other organs [3,4]. The most prevalent subtype is epithelial ovarian cancer, which is usually diagnosed at an advanced stage, when 5-year overall survival is only around 29%, in contrast with 92% for early-stage disease. The most common histological type of ovarian cancer is high-grade serous ovarian cancer (75% of all epithelial ovarian cancer patients). Part of the reason for the high mortality rate is that the disease is usually asymptomatic in its early stages and ovarian cancer only causes symptoms in its advanced stages that are not specific for the disease. The symptoms include abdominal bloating, early satiety, nausea, abdominal distension, change in bowel function, urinary symptoms, back pain, fatigue, and loss of weight, which typically present months before diagnosis [5].

The International Federation of Gynecology and Obstetrics (FIGO) staging system classifies patients with ovarian cancer into stages I to IV based on the size and extent of the tumor [5]. The primary treatment for ovarian cancer is primary debulking surgery with the goal of no residual disease, which means that less than 1 cm of tumor tissue remains after surgery (optimal/complete cytoreduction) [6]. After primer surgery, current guidelines recommend platinum-based adjuvant chemotherapy (carboplatin or cisplatin) in combination with taxane compounds (paclitaxel) [3,4,7]. If complete cytoreduction is considered impossible or has unacceptable preoperative morbidity, neoadjuvant chemotherapy (NACT) followed by interval debulking surgery (IDS) could be an alternative option [8]. Previous studies have shown the non-superiority of NACT + IDS compared to primary debulking surgery for the treatment of epithelial ovarian cancer, even if NACT + IDS treatment was associated with a significantly lower rate of postoperative complications [9].

Previous studies have shown that surgical cytoreduction is a strong predictor of prognosis, as a direct correlation exists between the extent of postsurgical tumor residuum and progression-free survival (PFS) and overall survival (OS) [4,5]. The theoretical advantage of surgical cytoreduction is that by removing large tumor masses, the host’s immune competence can be enhanced, thus preventing resistance to chemotherapy [4]. In some studies, high levels of tumor markers (human epididymis 4 (HE4) and cancer antigen-125 (Ca-125)) have been used as predictors of suboptimal debulking surgery [8]. A recent study by Feng et al. described the prognostic role of age, the preoperative serum Ca-125, and HE4 level in treatment planning for advanced-stage ovarian cancer patients. They suggested that these factors may be used as pre-evaluation before laparoscopy or diagnostic imaging prediction models [10]. Various studies have investigated the prognostic role of preoperative computed tomography (CT) imaging [8,11,12,13] and Ca-125 in predicting the feasibility of primary tumor reduction [8,13,14]. However, there is no clear guideline on exactly which criteria give patients a greater benefit from NACT + IDS [9].

Previous studies have found that calculated ratios of white blood cells [e.g., neutrophil-to-lymphocyte ratio (NLR), monocyte-to-lymphocyte ratio (MLR), and platelet-to-lymphocyte ratio (PLR)] determined from blood samples collected from patients prior to treatment have prognostic value for ovarian cancer [15]. Their independent predictive value of survival in epithelial ovarian cancer was assessed in multiple systematic reviews and meta-analyses [15,16,17]. It was observed that in patients with ovarian cancer, elevated NLR in peripheral blood samples taken before therapy (which correlated with elevated CA-125) was associated with a worse prognosis [18]. In the same setting, high PLR correlated with reduced OS and PFS [19]. The predictive value of these inflammatory biomarkers based on complete blood cell count for therapy selection at diagnosis of ovarian cancer has also been evaluated [15]. Preoperative NLR and PLR have been shown to predict platinum resistance in ovarian cancer [20].

The aim of our study was to investigate preoperative laboratory parameters of patients with serous epithelial ovarian cancer who underwent primary tumor reductive surgery in the Obstetrics and Gynecology Clinic of the University of Debrecen Clinical Centre between 2006 and 2013 and to evaluate the prognostic significance of these parameters in predicting complete or incomplete cytoreduction during primary surgery.

## 2. Materials and Methods

With the permission of the Ethics Committee of the University of Debrecen, we examined the clinical data from ovarian cancer patients who underwent primary surgery for tumor removal between 2006 and 2013 at the Obstetrics and Gynecology Clinic of the University of Debrecen Clinical Center. Selection criteria included the histological type of the tumor and only patients with serous epithelial ovarian cancer were included in our study. Patients were divided into two groups according to whether complete tumor reduction was achieved during primary surgery or whether a residual tumor greater than 1 cm remained.

The preoperative parameters we studied were: age, body mass index (BMI), stage (FIGO I-IV), total white blood cell count (WBC), absolute neutrophil granulocyte count (Neu#), absolute lymphocyte count (Ly#), absolute monocyte count (Mono#), platelet count (PLT), neutrophil-to-lymphocyte ratio (NLR = Neu#/Ly#), monocyte-to-lymphocyte ratio (MLR = Mono#/Ly#), platelet-to-lymphocyte ratio (PLR = PLT/Ly#), mean platelet volume (MPV), platelet distribution width (PDW), and Ca-125. In addition, data on progression-free survival (PFS) and overall survival (OS) were also collected.

Statistical calculations were performed with SPSS 25.0. Kolmogorov–Smirnov and Shapiro–Wilk tests were used as normality tests. Independent samples *t*-test was used to compare data between the two groups (complete vs. incomplete tumor reduction) in case of normal distribution of data, and the non-parametric Mann–Whitney U-test in case of non-normal distribution.

For those parameters that showed a significant difference between the complete and incomplete cytoreduction groups, we used receiver operating characteristic (ROC) curve analysis to examine whether they showed a correlation with the degree of cytoreduction achieved during PDS. For those parameters where a significant correlation was detected and the area under the curve was adequate, optimal cut-off values were determined based on Youden’s index, with corresponding sensitivity and specificity values.

Subsequently, we dichotomized the parameters at the defined cut-off values in the entire group of patients studied and performed a binary logistic regression model using the backward method, by which we examined the independent predictive value of the factors potentially predicting the achievement of complete tumor reduction.

Kaplan–Meier analysis was used to compare progression-free survival and overall survival curves between the two groups of patients.

A value of *p* < 0.05 was considered significant in the analyses.

## 3. Results

### 3.1. Clinical Data of Subjects

The clinical data of the patients in the study are shown in Table 1. During the study period, 150 patients with serous epithelial ovarian cancer underwent primary surgery, of whom 67 patients underwent successful tumor reduction and 83 patients underwent incomplete tumor reduction. The mean age of the patients was 57.11 years (SD = 12.40); 45 patients had FIGO stage I, 8 patients had FIGO stage II, 72 patients had FIGO stage III, and 25 patients had FIGO stage IV ovarian cancer; 67.6% of patients (97/150) were diagnosed with advanced stage (FIGO III-IV).

The Kolmogorov–Smirnov and Shapiro–Wilk tests showed that the age and BMI values of the patients were normally distributed. The WBC, Neu#, Ly#, Mono#, PLT, NLR, MLR, PLR, PDW, MPV, Ca-125, PFS, and OS values deviated from the normal distribution.

An independent sample *t*-test was used to compare the age and BMI of the two groups of patients (complete vs. incomplete tumor reduction). Non-parametric Mann–Whitney U test was used to compare WBC, Neu#, Ly#, Mono#, PLT, NLR, MLR, PLR, PDW, MPV, Ca-125, PFS, and OS values between the two patient groups (Table 1). These showed that the age (*p* < 0.01), WBC [U = 1838.00, Z = (−2.12), *p* < 0.05], Neu# [U = 1627.00, Z = (−2.89), *p* < 0.01], Mono# [U = 1499.00, Z = (−3.16), *p* < 0.01], PLT [U = 1697.00, Z = (−2.82), *p* < 0.01], NLR [U = 1343.50, Z = (−4.12), *p* < 0.001], MLR [U = 1262.00, Z = (−4.23), *p* < 0.001], PLR [U = 1245.00, Z = (−4.55), *p* < 0.001], and Ca-125 [U = 573.50, Z = (−3.87), *p* < 0.001] values of patients who underwent successful debulking surgery were significantly lower compared to patients with incomplete tumor reduction. There was no significant difference in BMI (*p* = 0.179) and MPV [U = 2189.00, Z = (−0.72), *p* = 0.46] between the two patient groups. PDW values [U = 1850.00, Z = (−2.17), *p* < 0.05] were significantly higher in the group of patients who underwent successful debulking surgery.

**Figure 1:**
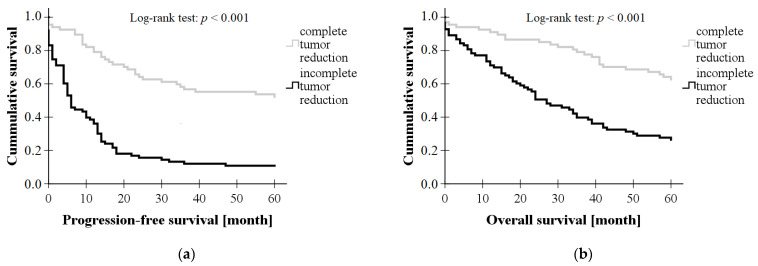
Comparison of (**a**) 5-year progression-free survival and (**b**) 5-year overall survival curves based on the outcome of primary tumor reduction surgery for patients with epithelial ovarian cancer (complete vs. incomplete tumor reduction during primary surgery).

Survival curves were plotted by Kaplan–Meier analysis and progression-free survival and overall survival were compared between the two patient groups using a log-rank test. Based on the results, both 5-year progression-free survival (*p* < 0.001, log-rank test) and overall survival (*p* < 0.001, log-rank test) were significantly higher in the group with complete tumor reduction; these results are shown in Figure 1.

### 3.2. ROC Curve Analysis

**Figure 2:**
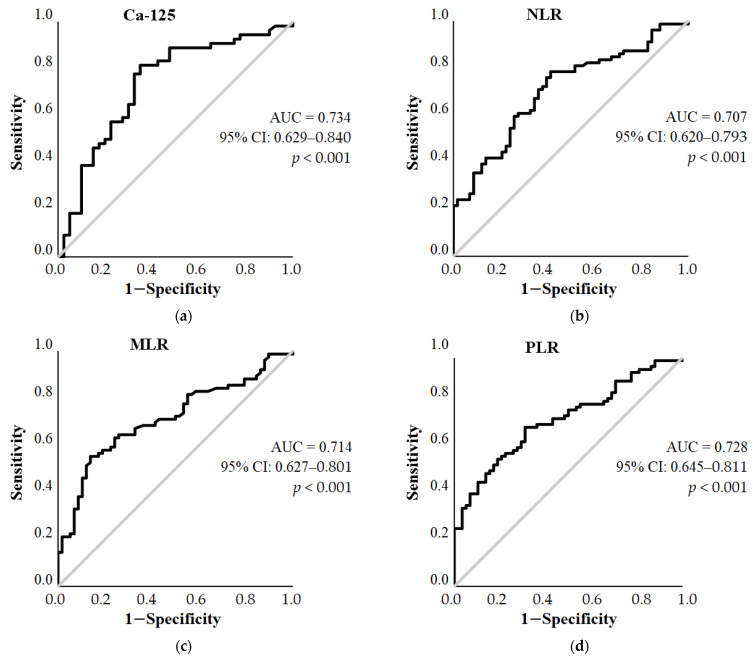

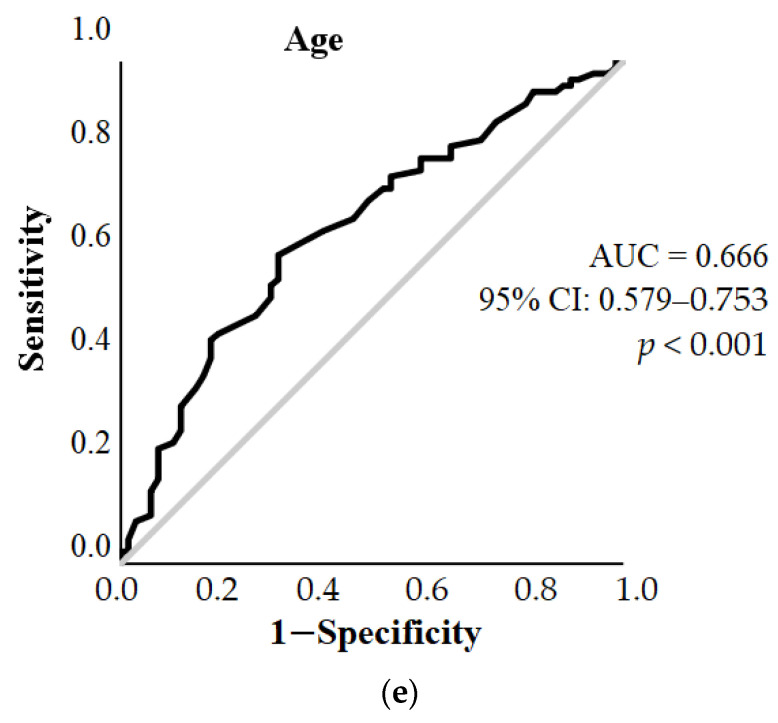
Receiver operating characteristic (ROC) curves of the predictors tested for predicting incomplete tumor reduction during primary surgery in serous epithelial ovarian cancer patients: (**a**) Ca-125; (**b**) neutrophil-to-lymphocyte ratio (NLR); (**c**) monocyte-to-lymphocyte ratio (MLR); (**d**) platelet-to-lymphocyte ratio (PLR); (**e**) age. AUC: area under the curve.

The optimal cut-off values for the outcome of the surgery were determined by ROC curve analysis. Variables with an area under the curve (AUC) greater than 0.7 were used as predictive models. Among the parameters we examined, for Ca-125, NLR, MLR, and PLR, we were able to establish a cut-off value for whether complete tumor reduction could be achieved during surgery (Figure 2).

Based on the results of ROC curve analysis, the optimal cut-off value for Ca-125 (AUC = 0.734, 95% CI: 0.629–0.840, *p* < 0.001) was 169.15 kU/L (sensitivity = 77.78%, specificity = 67.5%). The optimal cut-off value for NLR was 3.362 (AUC = 0.707, 95% CI: 0.620–0.793, *p* < 0.001, sensitivity = 70.89%, specificity = 63.79%), for MLR 0.305 (AUC = 0.714, 95% CI: 0.627–0.801, *p* < 0.001, sensitivity = 64.5%, specificity = 74.1%), and PLR 199.371 (AUC = 0.728, 95% CI: 0.645–0.811, *p* < 0.001, sensitivity = 69.62%, specificity = 68.97%). For age, the AUC was 0.666 (95% CI: 0.579–0.753, *p* < 0.001), with a cut-off of 57.5 years (sensitivity = 61.4%, specificity = 68.7%). The AUC values of WBC, Ly#, Neu#, Mono#, PLT, and PDW were 0.606 (95% CI: 0.507–0.704, *p* < 0.001), 0.679 (95% CI: 0.590–0.768, *p* < 0.001), 0.645 (95% CI: 0.549–0.741), 0.660 (95% CI: 0.567–0.753), 0.640 (95% CI: 0.548–0.732) 0.608 (95% CI: 0.511–0.706), respectively, and thus these parameters were not suitable for threshold setting.

### 3.3. Binary Logistic Regression

Using a binary logistic regression model, we examined the independent predictive value of factors potentially predicting the achievement of complete tumor reduction during primary surgery. In the statistical model, the variables were dichotomized according to the cut-off values obtained from the ROC curve; the parameters we considered were age (>57.5 years), FIGO stage (I-II vs III-IV), NLR (>3.362), MLR (>0.305), PLR (>199.371), and Ca-125 (>169.15 kU/L). The final step of the backward model includes only markers with independent predictive value, excluding non-significant and dependent factors (Table 2). The results of the logistic regression showed that among the parameters considered, stage [FIGO III-IV (OR = 5.102, 95% CI: 1.659–15.693, *p* < 0.01)], MLR [> 0.305 (OR = 5.028, 95% CI: 1.561–16.201, *p* < 0.01)], and Ca-125 [>169.15 (OR = 4.671, 95% CI: 1.540–14.167, *p* < 0.01)] were independent significant predictors of the degree of tumor reduction achieved during primary surgery.

**Figure 3:**
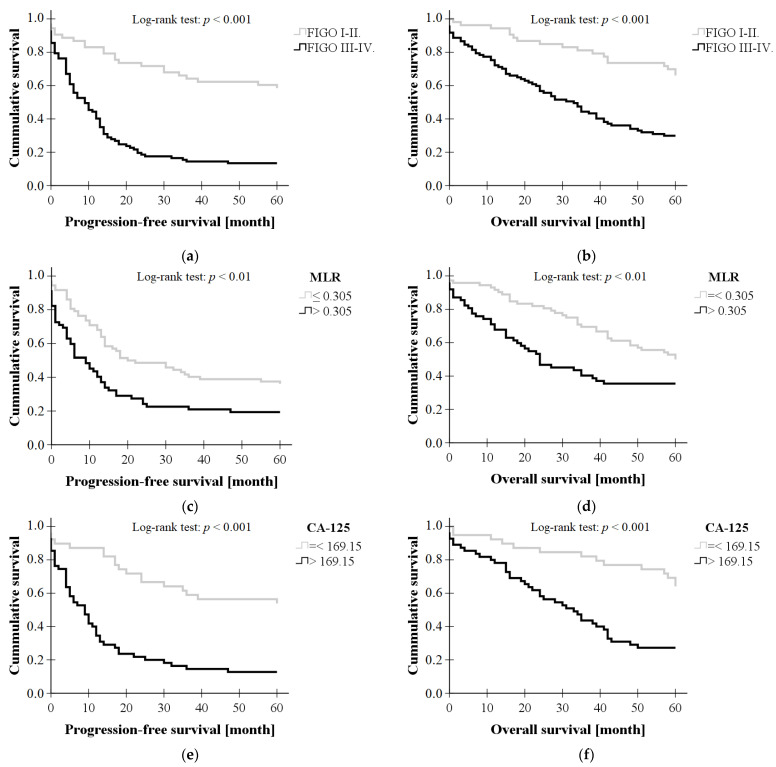
Progression-free survival and overall survival in various prognostic factors. (**a**,**b**) FIGO stage, (**c**,**d**) monocyte-to-lymphocyte ratio (MLR), and (**e**,**f**) Ca-125.

As shown in Figure 3, both the 5-year progression-free survival (*p* < 0.001, log-rank test) and overall survival rates (*p* < 0.001, log-rank test) were significantly higher in FIGO stage I-II patients compared to stage III-IV patients. The 5-year PFS and OS rates were also significantly higher for MLR ≤ 0.305 (*p* < 0.01, *p* < 0.01) and Ca-125 ≤ 169.15 kU/L (*p* < 0.001, *p* < 0.001).

**Figure 4:**
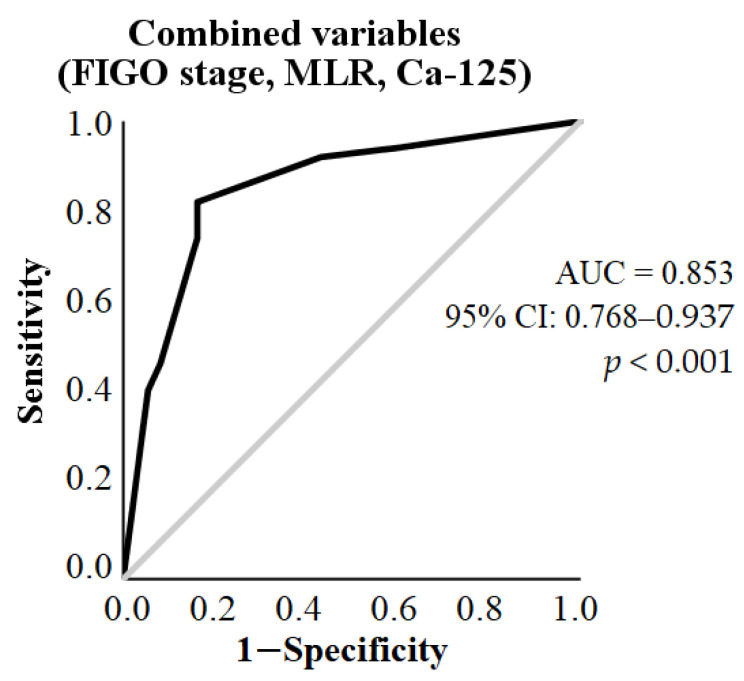
Analysis of the receiver operating characteristic (ROC) curve for combined predictors in predicting incomplete tumor reduction during primary surgery in patients with serous epithelial ovarian cancer. Predictors include FIGO stage III-IV, monocyte-to-lymphocyte-ratio (MLR) > 0.305, and Ca-125 > 169.15 kU/L, determined based on the last step of the binary logistic regression model. AUC: area under the curve.

Based on ROC curve analysis, the AUC with the combination of these parameters was 0.853 (95% CI: 0.768–0.937, *p* < 0.001) (Figure 4).

## 4. Discussion

Despite advances in treatment, ovarian cancer is still associated with a high mortality rate, and the rate of cytoreduction achieved during primary debulking surgery is a major prognostic factor for survival [4]. In our study, there was a significant difference between 5-year overall survival and progression-free survival of ovarian cancer patients undergoing primary debulking surgery depending on whether complete tumor reduction was achieved during primary surgery, which is consistent with the literature. However, there is no clear guideline on exactly which criteria give patients a greater benefit from NACT + IDS [9].

In recent times, several studies have been conducted to predict suboptimal cytoreduction in patients with ovarian cancer during primary surgery. Some studies in the literature have examined the predictive value of preoperative laboratory parameters, while others have examined data collected during imaging studies or diagnostic laparoscopy. According to the scoring system developed by Gu et al., the predictive index parameters related to a high risk of suboptimal cytoreduction include age > 60 years, CA-125 level > 800 U/mL, and several preoperative CT findings, such as abdominal bowel metastasis, spleen metastasis, diaphragmatic metastasis, and retroperitoneal lymph node enlargement above the inferior mesenteric artery. [21]. Piedimonte et al. developed a machine learning algorithm to predict the outcome of primary cytoreductive surgery in patients with advanced-stage ovarian cancer. Their study showed that the five most important contributors to the model to predict suboptimal cytoreduction were Ca-125, albumin, diaphragm disease, age, and ascites [22]. In a prospective study, Laios et al. aimed to develop an explainable artificial intelligence-based predictive model for complete cytoreduction of ovarian cancer [23].

Although a number of different approaches have been used in recent years to investigate the predictability of incomplete cytoreduction as part of the preoperative screening of ovarian cancer patients, several of these methods can be expensive or carry a higher risk for patients (diagnostic laparoscopy). Why, then, might preoperative inflammatory parameters play a role in predicting the success of primary debulking surgery?

In recent years, it has become clear that in ovarian cancer, there are several associations between immune function and the clinical course of the disease [3]. During carcinogenesis, the progenitor cell has to overcome a number of control processes and physiological imbalances, which it can achieve by different mechanisms. These mechanisms include independence from growth signals, insensitivity to growth inhibitory signals, unrestricted cell proliferation, reduced apoptosis, and the ability to vascularize, invade and metastasize [24]. It is now clear that tumors should be understood as organ-like structures in which there are complex bidirectional interactions between tumor-transformed and tumor-associated cells that form a stroma [25]. Cancer cells together with surrounding non-transformed cells form a tumor microenvironment (TME), the composition of which is a major determinant of cancer progression. The communication within the TME is controlled by a network of cytokines, growth factors, inflammatory mediators, and matrix remodeling enzymes [26]. The inflammatory mediators secreted by the transformed epithelial cells, such as cytokines, chemokines, growth factors, and prostaglandins, further enhance the development of an inflammatory environment, which may reprogram the surrounding cells to form a TME. The TME is composed mainly of endothelial cells, cancer-associated fibroblasts, adipocytes [26,27], and various immune cells, including myeloid-derived suppressor cells (MDSCs), tumor-associated macrophages, tumor-associated neutrophils, tumor-infiltrating lymphocytes (TILs) [26], and other cells that secrete additional growth factors and cytokines that enhance tumor progression [27]. The tumor microenvironment plays an important role in tumorigenesis and cancer cell growth [28].

As part of the cell-mediated adaptive immune response, T lymphocytes are thought to play an important role in tumor defense. Lymphocytes induce cytotoxic cell death and inhibit tumor cell proliferation and migration [29]. Studies have reported that the presence of CD3+ TILs and cytotoxic CD8+ TILs correlated with an improved clinical prognosis, while intratumoral CD4+ regulatory T cells, macrophages, and MDSCs were associated with a worse prognosis [3]. Elevated serum levels of interleukin (IL) -1β, IL-6, and IL-8 have been shown to correlate with worse prognosis in epithelial ovarian cancer [27].

Elements of the innate immune response such as macrophages, granulocytes, and natural killer (NK) cells have direct tumor-killing capacity through the production of mediators, but also have tumor progression-promoting effects [30]. Macrophages are phagocytic cells of the immune system that play a central role in inflammation, wound healing, and tissue repair [31]. They are activated from monocytes by monocyte chemoattractant protein-1 (MCP-1), cytokines (like IL-6), vascular endothelial growth factor (VEGF), and chemokines produced by neoplastic cells [32]. Monocytes produce numerous cytokines such as IL-6, IL-10, and IL-15 which contribute to the poor prognosis of tumors [33]. Furthermore, they differentiate into tumor-associated macrophages (TAMs) upon entering tumor tissue. Increased expression of TAMs in TMEs is also correlated with poor prognosis in malignant tumors [31]. TAMs with M2 phenotype can promote tumor survival by producing growth factors, promoting vascularization and tumor invasion, and inhibiting adaptive immune responses through cytokine and chemokine production [34]. Monocytes in peripheral blood may reflect the formation or presence of TAMs [29].

Tumor-associated neutrophils (TANs) have both pro- and antineoplastic functions, similar to macrophages [32]. Cancer cells play a vital role in stimulating the release of granulocyte colony-stimulating factors, which can increase the number of neutrophils. Neutrophil granulocytes are involved in tumor cell invasion and metastasis through the production of VEGF, proteases such as matrix metalloproteinase (MMP), and elastase enzymes [35].

In response to inflammation, platelets are activated, aggregate, and thrombocytosis develops [18]. Platelets are activated indirectly by tumor cells through tissue factor (TF). Activated platelets induce tumor angiogenesis through angiopoietin-1, VEGF, transforming growth factor ß, C-type lectin receptor 2, contribute to tumor metastasis induction through MMP, platelet-derived growth factor, induce tumor cell apoptosis through cytotoxic effect, and can promote tumor invasion [32]. It has been demonstrated that an increase in platelet numbers plays a role in tumor progression [31]. Thus, platelets also contribute to tumor growth, invasion, and tumor angiogenesis [28].

Systemic inflammation is associated with alterations in peripheral blood leukocytes [28]. The immune system and the inflammatory response play an important role in different stages of carcinogenesis such as initiation, invasion, promotion, and metastasis formation [35]. Overall, the presence of inflammation and inflammatory markers correlates with the prognosis of cancer, which allows their use as clinical prognostic markers [28]. Several studies have shown that inflammatory markers such as absolute neutrophil count, absolute monocyte count, absolute lymphocyte count, platelet count, and their calculated ratios such as NLR, MLR, and PLR are significantly associated with worse prognosis of several gynecological cancers [28]. Increasing evidence has shown that peripheral blood cells and relevant ratios (NLR, MLR, and PLR) are related to prognosis in ovarian cancer [16].

The purpose of our study was to assess the ability of preoperative laboratory parameters to predict the outcome of primary debulking surgery with regard to complete tumor reduction. There was a significant difference between the two groups of patients (complete vs. incomplete cytoreduction during surgery) in terms of age, absolute white blood cell counts, and their calculated values, NLR, PLR, MLR, and Ca-125 parameters.

Although a number of preoperative parameters showed significant differences between the complete and incomplete cytoreduction groups, the results of the ROC curve analysis showed that optimal cut-off values could be determined for only a few parameters. In our study, based on the results of ROC curve analysis, we found that age was significantly associated with the risk of suboptimal cytoreduction during primary debulking surgery, with a cut-off value of 57.5 years. Additionally, we were able to determine optimal cut-off values for incomplete cytoreduction using ROC curve analysis for NLR (cut-off value of 3.362), PLR (cut-off value of 199.371), CA-125 (cut-off value of 169.15 kU/L), and MLR. MLR was also found to be significantly associated with the risk of suboptimal cytoreduction during primary surgery, with a cut-off of 0.305. Above these thresholds, the chances of failing to achieve optimal cytoreduction during primary debulking surgery are therefore significantly increased.

The results of the binary logistic regression indicated that among the examined parameters MLR [with a cut-off value of 0.305 (3.28 if counted as LMR)] and Ca-125 (cut-off value of 169.15 kU/L) had a significant predictive value for predicting incomplete tumor reduction during primary surgery, in addition to disease stage.

Our results are in line with the results of a study by Eo. et al., who investigated preoperative parameters of epithelial ovarian cancer patients, such as FIGO stage, maximal diameter of residual tumor, ascites, serum albumin concentration, CA-125 levels, and hematologic parameters (peripheral blood WBC, hemoglobin, platelet counts, absolute neutrophil count, absolute monocyte count, absolute lymphocyte count, LMR, NLR, and PLR) that were obtained retrospectively. They also found a prognostic role for LMR, with a cut-off value of 3.75. Using multivariate logistic regression, age, CA-125, and LMR were found to be the strongest predictors for suboptimal cytoreduction. In their study, they investigated preoperative parameters of ovarian cancer patients with different histological types [36], whereas in our study, the serous epithelial subtype was specifically selected.

Finally, the ROC curve analysis using the three parameters (FIGO stage, preoperative MLR, and Ca-125) in combination showed a higher AUC compared to when the variables were tested separately. Based on this, we can conclude that the combined use of these parameters may help in estimating the risk of incomplete debulking surgery in the future.

Based on the results of Kaplan–Meier analysis (log-rank test), patients classified as stage I-II by FIGO staging had significantly higher PFS and OS compared to survival data for patients classified as stage III-IV. These results suggest that early diagnosis and determination of whether optimal cytoreduction is achievable is of paramount importance for the patient.

We also investigated the progression-free and overall survival of the patient groups divided according to the parameters obtained from binary logistic regression, using Kaplan–Meier analysis. In our study, the results of Kaplan–Meier analysis (log-rank test) demonstrated a significant difference in 5-year overall survival and progression-free survival of patients with serous epithelial ovarian cancer based on the parameters identified by binary logistic regression. Specifically, a preoperative MLR > 0.305, Ca-125 > 169.15 kU/L, and advanced stage of the disease (FIGO III-IV) were associated with poorer PFS and OS. These findings are consistent with previous analyses, which have demonstrated that low LMR predicts reduced OS and PFS and is associated with advanced FIGO stages, malignant ascites, lymph node metastasis, chemotherapy resistance, and high levels of Ca-125 [15,16,17].

It is important to acknowledge the limitations of our study, which include the non-randomized selection of patients into the two study groups and the retrospective nature of the data collection. These factors may have influenced the results obtained.

The significance of our results is that a blood test is a simple and inexpensive procedure that can be easily obtained during preoperative routine exams with a blood draw and can be repeated if necessary. In addition to classical clinicopathological factors such as stage, preoperative MLR, especially in combination with other parameters, can also be suitable to determine the prognosis. Moreover, in the future, MLR may help in selecting the best treatment option (primary debulking surgery or neoadjuvant chemotherapy + interval debulking surgery) for ovarian cancer patients with the greatest benefits. However, further prospective studies are needed to evaluate the optimal cut-off values of these markers for predicting optimal cytoreduction of serous epithelial ovarian cancer.

## 5. Conclusions

In our study, we found that the age, white blood cell, neutrophil, monocyte and platelet count, NLR, MLR, PLR, and Ca-125 values were significantly higher, while lymphocyte count and PDW were significantly lower in epithelial ovarian cancer patients who underwent primary debulking surgery with a suboptimal result. Using ROC curve analysis we estimated optimal cut-off values of Ca-125, MLR, NLR, PLR, and age for prediction of the incomplete cytoreduction. Based on the final step of the binary logistic regression model, the stage (FIGO III-IV), MLR, and Ca-125 were independent significant predictors of the degree of tumor reduction achieved during primary debulking surgery in ovarian cancer.

## Figures and Tables

**Table 1:** The clinical data of patients with serous epithelial ovarian cancer participating in the study.

		Complete Tumor Reduction (*n* = 67)	Incomplete Tumor Reduction (*n* = 83)	*p* Values
FIGO stage	I II	*n* = 35 *n* = 5 *n* = 22 *n* = 5	*n* = 10 *n* = 3 *n* = 50 *n* = 20	
	III			
	IV			
Age [year]		53.25 ± 11.88	60.22 ± 11.99	<0.01 *
BMI [kg/m^2^]		25.40 ± 5.27	26.56 ± 4.75	NS
WBC [G/L]		8.11 (3.99–20.43)	8.64 (4.88–19.49)	<0.05 *
Neu# [G/L]		5.25 (2.46–15.28)	6.21 (3.18–16.61)	<0.01 *
Ly# [G/L]		1.63 (0.77–4.20)	1.32 (0.39–11.62)	<0.001 *
Mono# [G/L]		0.38 (0.21–1.13)	0.49 (0.16–1.46)	<0.01 *
PLT [G/L]		314 (135.0–659.0)	348.5 (125.0–854.0)	<0.01 *
NLR		2.802 (0.99–9.35)	4.737 (0.377–34.56)	<0.001 *
MLR		0.232 (0.104–0.860)	0.361 (0.076–3.318)	<0.001 *
PLR		168.133 (78.512–428.571)	279.845 (21.084–1213.636)	<0.001 *
MPV [fl]		9.00 (7.40–12.0)	8.70 (7.0–12.30)	NS
PDW [fl]		44.40 (9.40–62.80)	39.90 (8.50–58.80)	<0.05 *
Ca-125 [kU/L]		81.20 (8.90–7915.0)	565.05 (8.40–6917.0)	<0.001 *

Mean values ± standard deviations (SD) or median (range) are presented. Complete tumor reduction: <1 cm of tumor tissue remains during tumor removal surgery. FIGO (International Federation of Gynecology and Obstetrics) staging: a system to classify the extent or spread of ovarian cancer as stage I–IV. (Stage I: Tumor confined to ovaries. II: Tumor involves 1 or both ovaries with pelvic extension (below the pelvic brim) or primary peritoneal cancer. III: Tumor involves 1 or both ovaries with cytologically or histologically confirmed spread to the peritoneum outside the pelvis and/or metastasis to the retroperitoneal lymph nodes. IV: Distant metastasis excluding peritoneal metastasis.) BMI: body mass index; WBC: white blood cell count; Neu#: absolute neutrophil granulocyte count; Ly#: absolute lymphocyte count; Mono#: absolute monocyte count; PLT: platelet count; NLR: neutrophil-to-lymphocyte ratio; MLR: monocyte-to-lymphocyte ratio; PLR: platelet-to-lymphocyte ratio; MPV: mean platelet volume; PDW: platelet distribution width. * Significant difference between values of patients who underwent complete vs. incomplete tumor reduction surgery. NS: not significant.

**Table 2:** The chance of incomplete tumor reduction during primary surgery in serous epithelial ovarian cancer patients.

	Odds Ratio	95% Confidence Interval	*p* Value
Stage (FIGO III-IV.)	5.102	1.659–15.693	<0.01
MLR > 0.305	5.028	1.561–16.201	<0.01
Ca-125 > 169.15	4.671	1.540–14.167	<0.01

FIGO: International Federation of Gynecology and Obstetrics, MLR: monocyte-to-lymphocyte ratio, CA-125 (kU/L).

## Data Availability

The data presented in this study are available on request from the corresponding author.
